# (2*R*)-2-Methyl­piperazinediium tetra­chloridocuprate(II)

**DOI:** 10.1107/S1600536810007877

**Published:** 2010-03-06

**Authors:** Li Zhaung Chen, Sheng Wan

**Affiliations:** a School of Material Science and Engineering, Jiangsu University of Science and Technology, Zhenjiang 212003, People’s Republic of China

## Abstract

In the title compound, (C_5_H_14_N_2_)[CuCl_4_], the copper(II) ion has a slightly tetra­hedrally distorted square-planar coordin­ation geometry and the diprotonated piperazine ring adopts a chair conformation. In the crystal structure, cations and anions are linked by inter­molecular N—H⋯Cl hydrogen bonds, forming a three-dimensional network.

## Related literature

For the ferroelectric and non-linear optical properties of chiral organic ligands, see: Fu *et al.* (2007 ▶); Qu *et al.* (2003 ▶). For transition metal complexes of 2-methyl­piperazine, see: Ye *et al.* (2009 ▶). For puckering parameters, see: Cremer & Pople (1975 ▶).
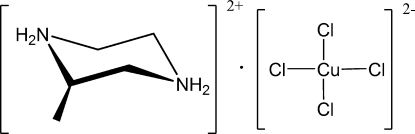


## Experimental

### 

#### Crystal data

(C_5_H_14_N_2_)[CuCl_4_]
*M* *_r_* = 307.53
Orthorhombic, 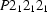

*a* = 6.0169 (12) Å
*b* = 12.985 (3) Å
*c* = 14.644 (3) Å
*V* = 1144.1 (4) Å^3^
*Z* = 4
Mo *K*α radiation
μ = 2.80 mm^−1^
*T* = 293 K
0.30 × 0.25 × 0.22 mm

#### Data collection

Rigaku SCXmini diffractometer
Absorption correction: multi-scan (*CrystalClear*; Rigaku, 2005 ▶) *T*_min_ = 0.87, *T*_max_ = 0.90
11992 measured reflections
2627 independent reflections
2469 reflections with *I* > 2σ(*I*)
*R*_int_ = 0.051

#### Refinement

*R*[*F* ^2^ > 2σ(*F* ^2^)] = 0.025
*wR*(*F* ^2^) = 0.060
*S* = 1.10
2627 reflections
111 parameters
1 restraint
H-atom parameters constrained
Δρ_max_ = 0.30 e Å^−3^
Δρ_min_ = −0.53 e Å^−3^
Absolute structure: Flack (1983 ▶), 1091 Friedel pairs
Flack parameter: 0.00 (3)

### 

Data collection: *CrystalClear* (Rigaku, 2005 ▶); cell refinement: *CrystalClear*; data reduction: *CrystalClear*; program(s) used to solve structure: *SHELXS97* (Sheldrick, 2008 ▶); program(s) used to refine structure: *SHELXL97* (Sheldrick, 2008 ▶); molecular graphics: *SHELXTL* (Sheldrick, 2008 ▶); software used to prepare material for publication: *SHELXL97*.

## Supplementary Material

Crystal structure: contains datablocks I, global. DOI: 10.1107/S1600536810007877/rz2412sup1.cif

Structure factors: contains datablocks I. DOI: 10.1107/S1600536810007877/rz2412Isup2.hkl

Additional supplementary materials:  crystallographic information; 3D view; checkCIF report

## Figures and Tables

**Table 1:** Hydrogen-bond geometry (Å, °)

*D*—H⋯*A*	*D*—H	H⋯*A*	*D*⋯*A*	*D*—H⋯*A*
N1—H1*A*⋯Cl1^i^	0.90	2.36	3.149 (6)	147
N1—H1*B*⋯Cl1^ii^	0.90	2.31	3.182 (6)	163
N2—H2*A*⋯Cl4^iii^	0.90	2.33	3.218 (6)	168
N2—H2*B*⋯Cl4^iv^	0.90	2.39	3.192 (6)	148

Symmetry codes: (i) 
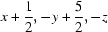
; (ii) 
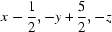
; (iii) 
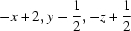
; (iv) 
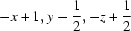
.

**Fig. 1.**
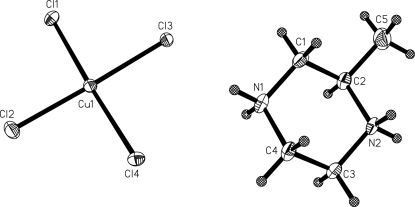
The asymmetric unit of the title compound with atom labels. Displacement ellipsoids were drawn at the 30% probability level.

**Fig. 2.**
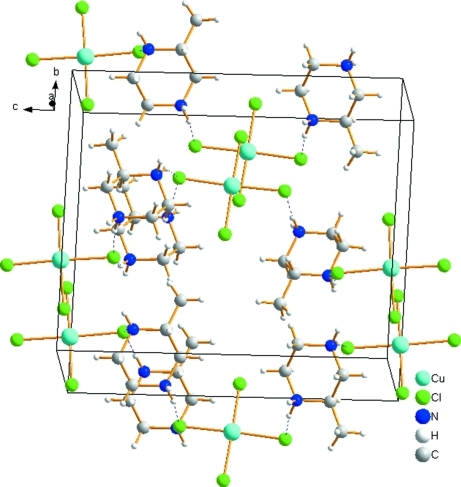
Packing diagram viewed approximately along the a axis. Hydrogen bonds are drawn as dashed lines.

## supplementary crystallographic information

### Comment

The existence of a chiral centre in an organic ligand is very important for the construction noncentrosymmetric or chiral coordination polymers that exhibit desirable physical properties such as ferroelectricity (Fu *et al.*, 2007) and nonlinear optical second harmonic generation (Qu *et al.*, 2003). Chiral (*R*)-2-methylpiperazine has a chiral centre which have shown tremendous scope in the synthesis of transition metal complexes (Ye *et al.*, 2009). The construction of new members of this family of ligands is an important direction in the development of modern coordination chemistry. We report here the crystal structure of the title compound

The asymmetric unit of the title compound consists of a diprotonated (*R*)-2-methylpiperazine cation and a tetrachlorocuprate anion (Fig. 1). The copper(II) metal centre is in a slightly tetrahedrally distorted square-planar coordination geometry (maximum displacement 0.0252 (18) Å for atom Cl1). The 6-membered piperazine ring adopts a chair conformation, with puckering parameters (Cremer & Pople, 1975) *Q* = 0.570 (6) Å, *θ* = 178.6 (6)° and *φ* = -127.3 (3)°. The crystal structure is stabilized by inter-ion N—H···Cl hydrogen interactions (Table 1) forming a three-dimensional network (Fig. 2).

### Experimental

A mixture of (*R*)-2-methylpiperazine (1 mmol, 0.1 g ), CuCl_2_ (1 mmol, 0.136 g) and 10% aqueous HCl (6 ml) were mixed and dissolved in 30 ml water by heating to 353 K (10 minute) forming a clear solution. The reaction mixture was then cooled slowly to room temperature. Single crystals of the title compound suitable for X-ray analysis were formed after 12 days on slow evaporation of the solvent.

### Refinement

All H atoms were placed in calculated positions with C—H = 0.93-0.98 Å, N—H = 0.90 Å, and refined using a riding model, with U_iso_(H) = 1.2U_eq_(C, N) or 1.5 U_eq_(C) for methyl H atoms.

### Crystal data

**Table:**

(C_5_H_14_N_2_)[CuCl_4_]	*F*(000) = 620
*M**_r_* = 307.53	*D*_x_ = 1.785 Mg m^−^^3^
Orthorhombic, *P*2_1_2_1_2_1_	Mo *K*α radiation, λ = 0.71073 Å
Hall symbol: P 2ac 2ab	Cell parameters from 2469 reflections
*a* = 6.0169 (12) Å	θ = 3.1–27.5°
*b* = 12.985 (3) Å	µ = 2.80 mm^−^^1^
*c* = 14.644 (3) Å	*T* = 293 K
*V* = 1144.1 (4) Å^3^	Block, yellow
*Z* = 4	0.30 × 0.25 × 0.22 mm

### Data collection

**Table:**

Rigaku SCXmini diffractometer	2627 independent reflections
Radiation source: fine-focus sealed tube	2469 reflections with *I* > 2σ(*I*)
graphite	*R*_int_ = 0.051
Detector resolution: 13.6612 pixels mm^-1^	θ_max_ = 27.5°, θ_min_ = 3.1°
ω scans	*h* = −7→7
Absorption correction: multi-scan (*CrystalClear*; Rigaku, 2005)	*k* = −16→16
*T*_min_ = 0.87, *T*_max_ = 0.90	*l* = −18→18
11992 measured reflections	

### Refinement

**Table:**

Refinement on *F*^2^	Hydrogen site location: inferred from neighbouring sites
Least-squares matrix: full	H-atom parameters constrained
*R*[*F*^2^ > 2σ(*F*^2^)] = 0.025	*w* = 1/[σ^2^(*F*_o_^2^) + (0.0239*P*)^2^ + 0.0175*P*] where *P* = (*F*_o_^2^ + 2*F*_c_^2^)/3
*wR*(*F*^2^) = 0.060	(Δ/σ)_max_ < 0.001
*S* = 1.10	Δρ_max_ = 0.30 e Å^−^^3^
2627 reflections	Δρ_min_ = −0.53 e Å^−^^3^
111 parameters	Extinction correction: *SHELXL97* (Sheldrick, 2008), Fc^*^=kFc[1+0.001xFc^2^λ^3^/sin(2θ)]^-1/4^
1 restraint	Extinction coefficient: 0.193 (9)
Primary atom site location: structure-invariant direct methods	Absolute structure: Flack (1983), 1091 Friedel pairs
Secondary atom site location: difference Fourier map	Flack parameter: 0.00 (3)

### Special details

**Table:**

Geometry. All esds (except the esd in the dihedral angle between two l.s. planes) are estimated using the full covariance matrix. The cell esds are taken into account individually in the estimation of esds in distances, angles and torsion angles; correlations between esds in cell parameters are only used when they are defined by crystal symmetry. An approximate (isotropic) treatment of cell esds is used for estimating esds involving l.s. planes.
Refinement. Refinement of *F*^2^ against ALL reflections. The weighted *R*-factor wR and goodness of fit *S* are based on *F*^2^, conventional *R*-factors *R* are based on *F*, with *F* set to zero for negative *F*^2^. The threshold expression of *F*^2^ > σ(*F*^2^) is used only for calculating *R*-factors(gt) etc. and is not relevant to the choice of reflections for refinement. *R*-factors based on *F*^2^ are statistically about twice as large as those based on *F*, and *R*- factors based on ALL data will be even larger.

### Fractional atomic coordinates and isotropic or equivalent isotropic displacement parameters (Å^2^)

**Table:**

	*x*	*y*	*z*	*U*_iso_*/*U*_eq_	
Cu1	0.82482 (13)	1.34903 (5)	0.01022 (5)	0.0237 (3)	
Cl4	0.8203 (3)	1.32659 (11)	0.17172 (9)	0.0251 (4)	
Cl3	0.8247 (3)	1.17476 (11)	−0.00811 (9)	0.0256 (4)	
Cl2	0.8317 (3)	1.52141 (11)	0.02454 (12)	0.0365 (4)	
Cl1	0.8196 (3)	1.36387 (11)	−0.14937 (10)	0.0250 (4)	
N1	0.8224 (9)	1.0679 (4)	0.1788 (3)	0.0289 (11)	
H1A	0.9338	1.1063	0.1559	0.035*	
H1B	0.6932	1.0976	0.1626	0.035*	
N2	0.6773 (9)	0.8919 (4)	0.2799 (3)	0.0263 (11)	
H2A	0.8089	0.8638	0.2951	0.032*	
H2B	0.5692	0.8523	0.3038	0.032*	
C5	0.6696 (17)	0.7855 (5)	0.1398 (5)	0.055 (2)	
H5C	0.5702	0.7410	0.1725	0.083*	
H5B	0.8190	0.7603	0.1458	0.083*	
H5A	0.6288	0.7867	0.0764	0.083*	
C2	0.6553 (12)	0.8933 (5)	0.1786 (4)	0.0300 (14)	
H2C	0.5097	0.9221	0.1630	0.036*	
C4	0.8395 (11)	1.0661 (5)	0.2797 (4)	0.0309 (14)	
H4B	0.8227	1.1354	0.3035	0.037*	
H4A	0.9850	1.0408	0.2975	0.037*	
C3	0.6623 (12)	0.9975 (5)	0.3195 (4)	0.0304 (13)	
H3B	0.6801	0.9939	0.3853	0.036*	
H3A	0.5170	1.0264	0.3067	0.036*	
C1	0.8335 (12)	0.9624 (5)	0.1390 (4)	0.0323 (14)	
H1D	0.9786	0.9329	0.1512	0.039*	
H1C	0.8150	0.9666	0.0733	0.039*	

### Atomic displacement parameters (Å^2^)

**Table:**

	*U*^11^	*U*^22^	*U*^33^	*U*^12^	*U*^13^	*U*^23^
Cu1	0.0339 (4)	0.0198 (4)	0.0174 (4)	−0.0001 (3)	−0.0003 (3)	0.0000 (3)
Cl4	0.0236 (7)	0.0309 (8)	0.0209 (7)	−0.0006 (7)	−0.0001 (7)	−0.0035 (6)
Cl3	0.0392 (8)	0.0197 (7)	0.0178 (7)	−0.0004 (7)	0.0001 (7)	0.0001 (5)
Cl2	0.0540 (10)	0.0222 (8)	0.0333 (9)	−0.0006 (8)	−0.0017 (10)	−0.0016 (6)
Cl1	0.0238 (7)	0.0308 (8)	0.0205 (7)	−0.0010 (7)	−0.0001 (7)	0.0047 (6)
N1	0.025 (2)	0.034 (3)	0.028 (3)	−0.004 (3)	0.000 (3)	0.013 (2)
N2	0.026 (2)	0.028 (3)	0.024 (3)	−0.004 (3)	0.002 (3)	0.011 (2)
C5	0.090 (6)	0.035 (4)	0.041 (5)	−0.005 (5)	−0.005 (6)	0.000 (3)
C2	0.033 (3)	0.031 (3)	0.026 (3)	−0.001 (3)	−0.003 (3)	0.007 (3)
C4	0.037 (3)	0.031 (3)	0.025 (3)	−0.004 (3)	−0.003 (3)	0.009 (3)
C3	0.037 (3)	0.032 (3)	0.023 (3)	0.001 (3)	0.005 (3)	0.006 (3)
C1	0.037 (3)	0.039 (4)	0.022 (3)	0.000 (3)	0.005 (3)	0.007 (3)

### Geometric parameters (Å, °)

**Table:**

Cu1—Cl2	2.2485 (16)	C5—H5C	0.9600
Cu1—Cl3	2.2788 (16)	C5—H5B	0.9600
Cu1—Cl1	2.3451 (15)	C5—H5A	0.9600
Cu1—Cl4	2.3831 (15)	C2—C1	1.514 (9)
N1—C4	1.482 (8)	C2—H2C	0.9800
N1—C1	1.490 (8)	C4—C3	1.506 (9)
N1—H1A	0.9000	C4—H4B	0.9700
N1—H1B	0.9000	C4—H4A	0.9700
N2—C2	1.489 (8)	C3—H3B	0.9700
N2—C3	1.492 (7)	C3—H3A	0.9700
N2—H2A	0.9000	C1—H1D	0.9700
N2—H2B	0.9000	C1—H1C	0.9700
C5—C2	1.513 (9)		
			
Cl2—Cu1—Cl3	178.25 (7)	N2—C2—C5	111.0 (5)
Cl2—Cu1—Cl1	90.66 (6)	N2—C2—C1	109.0 (5)
Cl3—Cu1—Cl1	87.95 (5)	C5—C2—C1	111.4 (6)
Cl2—Cu1—Cl4	91.68 (6)	N2—C2—H2C	108.5
Cl3—Cu1—Cl4	89.74 (5)	C5—C2—H2C	108.5
Cl1—Cu1—Cl4	177.28 (6)	C1—C2—H2C	108.5
C4—N1—C1	111.9 (4)	N1—C4—C3	110.3 (6)
C4—N1—H1A	109.2	N1—C4—H4B	109.6
C1—N1—H1A	109.2	C3—C4—H4B	109.6
C4—N1—H1B	109.2	N1—C4—H4A	109.6
C1—N1—H1B	109.2	C3—C4—H4A	109.6
H1A—N1—H1B	107.9	H4B—C4—H4A	108.1
C2—N2—C3	111.8 (4)	N2—C3—C4	110.5 (5)
C2—N2—H2A	109.3	N2—C3—H3B	109.6
C3—N2—H2A	109.3	C4—C3—H3B	109.6
C2—N2—H2B	109.3	N2—C3—H3A	109.6
C3—N2—H2B	109.3	C4—C3—H3A	109.6
H2A—N2—H2B	107.9	H3B—C3—H3A	108.1
C2—C5—H5C	109.5	N1—C1—C2	111.3 (5)
C2—C5—H5B	109.5	N1—C1—H1D	109.4
H5C—C5—H5B	109.5	C2—C1—H1D	109.4
C2—C5—H5A	109.5	N1—C1—H1C	109.4
H5C—C5—H5A	109.5	C2—C1—H1C	109.4
H5B—C5—H5A	109.5	H1D—C1—H1C	108.0
			
C3—N2—C2—C5	−179.7 (7)	N1—C4—C3—N2	56.1 (7)
C3—N2—C2—C1	57.3 (7)	C4—N1—C1—C2	56.4 (7)
C1—N1—C4—C3	−55.8 (7)	N2—C2—C1—N1	−56.0 (7)
C2—N2—C3—C4	−58.1 (7)	C5—C2—C1—N1	−178.8 (6)

### Hydrogen-bond geometry (Å, °)

**Table:**

*D*—H···*A*	*D*—H	H···*A*	*D*···*A*	*D*—H···*A*
N1—H1A···Cl1^i^	0.90	2.36	3.149 (6)	147
N1—H1B···Cl1^ii^	0.90	2.31	3.182 (6)	163
N2—H2A···Cl4^iii^	0.90	2.33	3.218 (6)	168
N2—H2B···Cl4^iv^	0.90	2.39	3.192 (6)	148

Symmetry codes: (i) *x*+1/2, −*y*+5/2, −*z*; (ii) *x*−1/2, −*y*+5/2, −*z*; (iii) −*x*+2, *y*−1/2, −*z*+1/2; (iv) −*x*+1, *y*−1/2, −*z*+1/2.
